# Acknowledgement to Reviewers of *Nutrients* in 2016

**DOI:** 10.3390/nu9010060

**Published:** 2017-01-12

**Authors:** Nutrients Editorial Office

**Affiliations:** MDPI AG, St. Alban-Anlage 66, 4052 Basel, Switzerland; nutrients@mdpi.com

The editors of *Nutrients* would like to express their sincere gratitude to the following reviewers for assessing manuscripts in 2016.

We greatly appreciate the contribution of expert reviewers, which is crucial to the journal’s editorial process. We aim to recognize reviewer contributions through several mechanisms, of which the annual publication of reviewer names is one. Reviewers receive a voucher entitling them to a discount on their next MDPI publication and can download a certificate of recognition directly from our submission system. Additionally, reviewers can sign up to the service Publons (https://publons.com) to receive recognition. Of course, in these intiatives we are careful not to compromise reviewer confidentiality. Many reviewers see their work as a voluntary and often unseen part of their role as researchers. We are grateful to the time reviewers donate to our journals and the contribution they make.

If you are interested in becoming a reviewer for *Nutrients*, see the link at the bottom of the webpage http://www.mdpi.com/reviewers.

The following reviewed for *Nutrients* in 2016:
Aadland, EivindHarisa, Gamaleldin I.Pastor-Valero, MariaAakre, IngerHarris, GabrielPatel, Ravi MangalAas, Turid SynnøveHarris, MargaretPaulsen, Berit SmestadAbbott, David H.Harris, WilliamPazdro, RobertAbe, SumikoHarrison, FionaPeacock, AmyAbenavoli, LudovicoHartman-Petrycka, MagdalenaPearce, Elizabeth N.Abeti, RosellaHasegawa, GojiPedraza-Chaverri, JoséAboud, FrancesHashem, Kawther M.Peiffer, JeremiahAbrahams, PeterHashimoto, NaotoPekkinen, MinnaAbreu, SandramrHatfield, DolphPeleg-Raibstein, DariaAceti, AriannaHattori, YuichiPeluso, IlariaAcuña, DaríoHaub, MarkPence, BrandtAdam, Clare L.Hawley, JohnPeng, Chiung–HueiAdams, JamesHayakawa, YoshihiroPenugonda, KavithaAdams, Sean H.Hayward, JoshuaPeoples, Gregory E.Afman, LydiaHeaney, Susan E.Pepino, M. YaninaAfzal, MohammedHearst, Mary O.Pereira, DoraAggarwal, AbhishekHeber, DavidPereira-da-Silva, LuisAgmon-Levin, NancyHébert, James R.Perera, Liyanage P.Agostoni, CarloHecht, Jacqueline T.Pérez-Berezo, TeresaAhmad, NihalHector, Amy J.Pérez-Fernández, VirginiaAigner, ElmarHedrick, ValisaPerez-Jimenez, JaraAkatsu, HiroyasuHegazi, Refaat A.Perna, AlessandraAkkermans, Marjolijn D.Heianza, YorikoPerng, WeiAl Subhi, LyuthaHeil, Sandra G.Perrella, Sharon L..Alaedini, ArminHeindel, JerroldPerrine, Cria G.Alagiakrishnan, KannayiramHeinrich, KatiePerry, TracyAlaofe, HalimatouHellgren, LarsPerticone, FrancescoAlashi, Adeola MHelms, Eric R.Pes, Giovanni M.Albano, EmanueleHemilä, HarriPeters, Rachel L.Alcorn, Joseph L.Henagan, Tara M.Peterson, Amie L.Aldini, GiancarloHendrich, SuzannePeterson, JuliaAl-Dujaili, EmadHendrie, GillyPeterson, LindaAlexandre-Gouabau, Marie-cécile F.Henein, MichaelPetrick, Jessica L.Alexy, UteHenjum, SigrunPfeifer, MichaelAlhazmi, AmaniHenkin, Ya'akovPfeuffer, MariaAlisi, AnnaHennessy, ÁinePhillips, LindaAlizadehkhaiyat, OmidHennigar, StevePhillips, StuartAllegaert, KarelHéon, MarjolainePhung, OliviaAllen, AndrewHernandez, J. LuisPiccolo, Brian D.Alleva, RenataHernández-Hernández, AngelPierzynowski, Stefan G.Allgrove, JudithHerrero, LauraPifferi, FabienÁlvarez, Leónides FernándezHerrmann, WolfgangPignitter, MarcAlwahsh, Salamah M.Heuberger, Roschelle A.Pignotti, GiselleAlway, Stephen E.Hew-Butler, TamaraPiknova, BarboraAlzaid, FawazHilmers, AngelaPilutti, Lara A.Amaravadi, Ravi K.Himotoe, TakashiPinkaew, SiwapornAmeri, PietroHinson, Andrew M.Pino-Figueroa, AlejandroAmes, NancyHinton, Pamela S.Pipingas, AndrewAmita, VaidyaHirasawa, AkiraPirisi-Creek, LuciaAn, RuopengHirsch, SandraPissios, PavlosAnderson, Emma L.Ho, Chi-TangPitts, Stephanie JilcottAnderson, Rozalyn M.Ho, MandyPizarro, FernandoAndjelkovic, Anuska V.Ho, Tin-YunPoff, Angela M.Andrade-Cetto, AdolfoHobin, ErinPolesel, JerryAndressoo, Jaan-OlleHocher, BertholdPolidori, Maria CristinaAnguah, KathereneHodge, AllisonPollán, MarinaAngulo, JavierHodgson, JonathanPoluzzi, ElisabettaAnnunziata, AzzurraHoeller, UlrichPons, AntoniAnsorena, DianaHoevenaar-Blom, Marieke P.Poole, David C.Anthony, TracyHoffman, MariaPoole, Jill A.Antoine-Jonville, SophieHoffman, RichardPopat, AmiraliAnton, Stephen D.Holder, AlvinPoppitt, SallyAnton, SteveHolick, Michael F.Porter, Judi A.Aoi, WataruHolliday, Elizabeth G.Posovszky, CarstenAppleton, KatherineHollis, BrucePotischman, NancyAprea, EugenioHolst, Jens JuulPoudyal, HemantAraújo, JoanaHoman, PhilippPourshahidi, KirstyAraujo, Joao RicardoHong, Mee YoungPowles, John W.Arcan, ChrisaHoon, Matthew W.Praagman, JaikeArciero, PaulHoppu, UllaPrasad, SahdeoArcot, JayashriHorio, YoshiyukiPrimeaux, Stefany D.Arguelles, SandroHorn, Susan D.Prior, Jerilynn C.Aris, RobertHorne, Benjamin D.Probert, ChrisArmstrong, Gavin R.Horrigan, Louise A.Probst, YasmineArosio, PaoloHossain, ZakirProdam, FlaviaArribas, Silvia M.Hou, Wen-ChiPulido, Olga M.Arshad, AliHoughton, LisaPullar, JulietArulselvan, PalasamyHouston, Mark C.Puri, Basant K.Arvidsson, LouiseHowarth, GordonPursey, KirrillyAschemann-Witzel, JessicaHowe, AnnaQi, DaiAstorino, ToddHrelia, PatriziaQian, QiAthyros, VasiliosHsiang, Chien-YunQin, BoAtkins, Janice L.Hsieh, Ching HuaQin, WeiAtkinson, SarahHsieh, Shu-ChenQuigley, EamonAttoub, SamirHu, Ming-ChangRaatz, Susan K.Augustin, HannaHu, ShenRabasa-Lhoret, RemiAugustin, LiviaHu, TianRabinowitz, Joshua D.Augustin, MaryHuang, Chi-ChangRadmacher, PaulaAura, Anna-MarjaHuang, Tzou-ChiRai, Dilip K.Auricchio, SalvatoreHughes, Dr. DavidRainger, G. EdAutier, PhilippeHugo Teixeira, VitorRajapakse, Niwanthi W.Avery, JodieHulsegge, GerbenRajendran, PraveenAxelsson, JohnHulston, CarlRamadori, GiulianoAzcárate-Peril, M. AndreaHulthén, LenaRamer-Tait, AmandaAzmi, Asfar SohailHunsberger, MonicaRamkumar, VickramBabu, DineshHunt, JanetRamos, SoniaBae, Jong-MyonHur, YangimRangan, AnnaBaek, Sang HongHurst, NancyRao, U. SubrahmanyeswaraBailey, StephenHursthouse, AndrewRaqib, RubhanaBaïz, NourHutchins, AndreaRasmussen, Heather E.Baker, JulienHuth, PeterRatamess, NicholasBaksh, ShairazHuysentruyt, KoenRatnayaka, J. ArjunaBanerjee, JheelamHwang, In KooRay, UdayanBanik, Sudip DattaHwang, Kyung-AReboul, EmmanuelleBao, YongpingHyun, Kyung-YaeRegenold, William T.Baragetti, AndreaIacobazzi, VitoRegina, Brigelius-FloheBaraldi, MarioIametti, StefaniaRegnault, TimothyBaranowski, TomIbarra, AlvinRegula, JulitaBarbara, AltieriIchii, HirohitoRehm, ColinBarbaresko, JanettIglesia, IrisReichmann, FlorianBarbonetti, ArcangeloIha, HidekatsuReicks, MarlaBarbour, JayneImperatori, ClaudioReidlinger, DianneBarclay, DenisIndrio, FlaviaReidy, PaulBarille-Nion, SohieInman, SharonReifen, RamBarker, TylerIqbal, KhalidReimer, RayleneBarnett, AliciaIriti, MarcelloReiner, MartinBarnett, Matthew P.G.Irshad, ShaziaReiser, JochenBarnhill, KellyIshitsuka, YoichiReisman, Scott A.Baron, KellyIshmael, Jane E.Reitelseder, SørenBarrero, AnnaIslam, Md SorifulRemacha, Ángel F.Barth-Jaeggi, TanjaIsmail, RitaRembialkowska, EwaBartlett, JonIspoglou, TheocharisRenner, UlrichBasak, Saroj K.Issaka, Abukari I.Renström, FridaBasu, ArpitaItamar, RazRespino, MatteoBateman, EmmaIuliano, SandraRESTANI, PatriziaBates, ChristopherIvy, John L.Reverri, ElizabethBatista Jr., Miguel L.Iwamori, MasaoRevuelta-Iniesta, RaquelBattino, MaurizioJaacks, Lindsay M.Rey, GuillaumeBaum, JamieJaakkola, JohannaReyes, MoraymaBaum, ThomasJabbarzadeh, EhsanReyners, Anna K.L.Baxter, SuzanneJack, SusanRhyu, Mee-RaBayat, ArezouJackman, SarahRice, Madeline MurguiaBayry, JagadeeshJackson, PhilippaRichter, JörgBazzano, AlessandraJacobs Jr., David R.Riddell, LynnBeck, Kathryn L.Jacobs, David R.Riedel, Christian U.Beckett, Emma LouiseJacobs, ReneRiley, MalcolmBeekley, MatthewJacobsen, RamuneRios, Jose LuisBei, RobertoJacobson, KevanRios-Covian, DavidBelay, BrookJadavji, Nafisa M.Robert-McComb, Jacalyn J.Bellar, DavidJadeja, RavirajsinhRoberts, Michael W.Bellisle, FranceJaeger, RalfRoberts, RichardBeloqui, AnaJaeger, WalterRobertson, M. DeniseBenamouzig, RobertJafariNasabian, PegahRobinson, LaurenBenatar, Jocelyne R.Jahan-Mihan, AlirezaRobinson, NedBendtsen, Line Q.Jahns, LisaRobles, BrendaBenn, Christine StabellJakobsen, JetteRobson, Shannon M.Benzeroual, Kenza E.Jakubowicz, DanielaRoche, EnriqueBergamin, MarcoJalili, ThunderRodrigues, Luís PauloBergdahl, Celena SchedeJamieson, Jennifer A.Rodriguez Artalejo, FernandoBergman, PeterJamurtas, AthanasiosRodriguez-Rodriguez, ElenaBergö, MartinJanaswamy, SrinivasRoelofs, EricaBermingham, EmmaJean, GuillaumeRogers, LisaBernstein, PaulJeewon, RajeshRoggero, PaolaBerry, NarelleJeffery, Elizabeth H.Roh, Gu SeobBevilacqua, Maria AssuntaJenkins, DavidRohner, FabianBhatia, JatinderJenkins, David J.A.Rohrmann, SabineBhupathiraju, ShilpaJensen, JørgenRomán, Gustavo C.Bhutta, Zulfiqar A.Jensen, Morten GeorgRomano, ClaudioBiagi, FedericoJeon, Sung HoRomero, Francisco JavierBiasutto, LuciaJiao, LiRondanelli, MariangelaBidel, Siamak BidelJin, RanRoot, Martin M.Biesiekierski, JessicaJoffre, CorinneRose, DevinBijak, MichałJohnson, ElizabethRosendale, Douglas IanBilleter, AdrianJohnson, IanRosenlund, HelenBinia, AristeaJohnson, Sarah A.Rosinger, AsherBirt, Diane F.Johnson, Stuart K.Rosner, BernardBitto, AlessandraJohnston, CarolRossi, FilippoBjørke-Monsen, Anne LiseJohnstone, Alexandra M.Rossi, MauroBlachier, FrançoisJoles, JaapRossi, MiriamBlack, KatherineJolliffe, David A.Rossi, Roberta ElisaBlack, LucindaJones, Daniel R.Rostami, KamranBlaner, WilliamJones, KerryRousseau, GuyBlanton, CynthiaJoo, Nam-seokRowlands, David S.Blomstrand, EvaJordan, IrmgardRoy, AditiBoaz, MonaJoseph, JacobRudkowska, IwonaBoers, HannyJoshipura, KaumudiRühl, RalphBöhm, VolkerJoubert, MichaelRuiz-Canela Lopez, MiguelBohn, TorstenJoven, JorgeRumbold, PennyBolaños-Ríos, PatriciaJudge, Michelle P.Rune, Gabriele M.Bolling, Bradley W.Jugdaohsingh, RavinRusso, MariaBoncinelli, FabioJung, SeungyounRyan, LisaBondonno, CatherineJunior, Walter A.R.Ryckman, Kelli K.Bondonno, CathyJuonala, MarkusRyffel, BernhardBonilha, Heather S.Jurewitsch, BrianRyu, Jong HoonBonnefont-Rousselot, DominiqueJurjus, Rosalyn A.Sacchetti, LuciaBooth, AlisonKabbani, Toufik A.Sacks, Frank M.Boquien, Clair-YvesKadi, FawziSadanaga, TsuneakiBordoni, AlessandraKafatos, AnthonySadoshima, JunichiBorradaile, Nica M.Kagawa, MasaharuSafouris, ApostolosBosch-Barrera, JoaquimKaidonis, GeorgiaSahu, Saura C.Boshuizen, HendriekKaikkonen, JariSaisho, YoshifumiBoucher, Barbara J.Kakigi, RyoSaito, IsaoBoué, StephenKakulas, FoteiniSakhaee, KhashayarBouhlal, SofiaKalhan, SatishSakkas, Giorgos K.Boulé, NormandKalman, DougSalgado, RobertoBoutcher, StephenKalsbeek, AndriesSalim, SaminaBowen, PhyllisKaludercic, NinaSalis, AmandaBoyle, VeronicaKamada, NobuhikoSalisbury, ElisabethBoztuğ, YaseminKamolz, LarsSalles, ChristianBrain, Susan D.Kanai, MasashiSalomone, FedericoBranco, MiguelKandzari, Stanley J.Salvatore, FrancescoBrandhagen, MartinKang, HeeSambataro, MariaBrand-Miller, JennieKang, J. YongSamieri, CéciliaBrandon, AmandaKang, Jae SeungSamuelsen, Anne Berit C.Brantsæter, Anne LiseKang, Jae-HeonSantamaria, RitaBravi, FrancescaKang, Ki SungSantangelo, CarmelaBravo, LauraKano, MitsuyoshiSantarpia, LidiaBrazzale, Danny J.Kaplan, Bonnie J.Santoro, AureliaBreen, CathyKappen, ClaudiaSantoro, DomenicoBriand, LoicKapsokefalou, MariaSantos, DianaBroderick, Tom L.Karachaliou, MariannaSantos-Gallego, CarlosBrookes, Denise S.K.Karagiannis, TomSantulli, GaetanoBrooks, HeatherKaraki, Shin-IchiroSapone, AnnaBrosnihan, K. BridgetKarakochuk, CrystalSardao, VilmaBrottveit, MargitKarayannakidis, PanayotisSarmento, BrunoBrough, LouiseKarras, Spiros N.Sarriá, BeatrizBrown, AndrewKarunasinghe, NishiSarris, JeromeBrown, LindsayKasai, HiroshiSatija, AmbikaBrown, RachelKasiotis, Konstantinos M.Sato, KenjiBrownlee, IainKasmi, Karim C.E.L.Sato, ShinBrownlee, Iain A.Kato, HisanoriSauder, Katherine AnnBrozek, WolfgangKatz, YitzhakSavaraj, NiramolBuckland, Nicola J.Katzmarzyk, Peter T.Savaskan, Nicolai E.Buechler, ChristaKavouras, StavrosSayón-Orea, CarmenBügel, SusanneKawai, YoshichikaSbrana, CristianaBuhling, Kai J.Kayano, Shin-ichiScazzina, FrancescaBührer, ChristophKazlauskaite, RasaSchalinskie, KevinBullon, PedroKeely, SimonSchapowal, AndreasBuonocore, DanielaKeku, TemitopeScharf, Rebecca J.Burke, Rachel M.Kelleher, CecilySchatten, HeideBurkhart, SarahKendall, CyrilSchauss, AlexBurmeister, JacobKennedy, Brian K.Scheers, Nathalie M.Burrows, TracyKenney, Erica L.Scheffler, ChristianeBurton-Freeman, BrittKern, Timothy S.Scherf, KatharinaBuschini, AnnamariaKeshteli, Ammar H.Schievano, ElisabettaBusik, Julia V.Key, Tim J.Schilling, JoelBustamante, SoniaKhalil, AbdelouahedSchipper, LidewijButeau, JeanKhosla, PramodSchlueter, NadineButriss, JudithKiely, MaireadSchmetterer, LeopoldByrne, RebeccaKietzmann, ThomasSchmid, Sebastian M.Cadario, FrancescoKim, Bong-SungSchmidt, Julie A.Cai, HuiKim, Byung JooSchmoeckel, JulianCai, LuKim, Chang-OSchoeller, DaleCairo, GaetanoKim, Heui-SooSchoenfeld, Brad J.Calaf, Gloria M.Kim, JaehanScholes, ShaunCalapai, GioacchinoKim, Kil-SooScholz-Ahrens, KatharinaCalder, PhilipKim, Kyung-suSchröder, KatrinCaldwell, Kathleen L.Kim, Mi KyungSchroeder, HelmutCalò, LeonardoKim, Na-HyungSchulze, KerryCalviello, GabriellaKim, Reuben H.Schwab, Charles G.Cambras, TrinitatKim, Sung-HoonSchwarz, PeterCamhi, SarahKim, Young-WooSclafani, AnthonyCamilleri, MichaelKim, Yun-baeScott, DavidCamire, MaryKim, YuriScott, SamCampbell, Sara ChellandKing, NeilScudiero, OlgaCamporeale, AnnalisaKingsley, MichaelSeal, JudyCannell, John J.Kipp, Anna PatriciaSebekova, KatarinaCansby, EmmelieKirkpatrick, Sharon I.Seca, Ana M.L.Cantorna, MargheritaKishimoto, YoshimiSechi, GianPietroCapelli, CarloKitic, Cecilia MarySeehaus, StefanieCarbone, John W.Kitson, AlexSeeman, Mary V.Carbone, MicheleKitson, Matthew T.Segovia-Siapco, GinaCardillo, CarmineKlaus, SusanneSeigler, DavidCarlberg, CarstenKleinewietfeld, MarkusSekikawa, AkiraCaroli, Anna M.Knapik, JosephSengupta, BidishaCarpéné, ChristianKneepkens, C. M. FrankSerra, DolorsCarpenter, GuyKnight, ChristineSerra, Monica C.Carroccio, AntonioKnox, Barbara StewartSéverine, LamonCasanovas, CarmenKo, Jiunn-LiangSha, ZheCases, JulienKoch, ManjaShahab-Ferdows, SettiCaspersen, Ida H.Koh, Gar YeeShahar, DanitCastell, Lindy M.Kojima, ItaruShaikh, Mohd. FarooqCastro-Cabezas, ManuelKojima-Yuasa, AkikoShaikh, Saame RazaCatenacci, Victoria A.Kojo, ShosukeShakibaei, MehdiCauli, OmarKones, RichardShamah-Levy, TeresaCedernaes, JonathanKonishi, HirotakaShao, AndrewCelton-Morizur, SeverineKontoyianni, MeropiSharma, BheshCengotitabengoa, MonicaKoo, Winston W.K.Sharma, NaveenCeolotto, GiulioKorenromp, Eline L.Sharma, SushilCercamondi, ColinKornprat, PeterShea, KylaCeresini, GrazianoKorte, AlexanderShea, TomCerletti, ChiaraKosińska, AgnieszkaShen, LeslieCesare Marincola, FlaminiaKostner, Gerhard M.Shen, Szu-ChuanCetin, IreneKotsakis, Georgios A.Sherman, Stephanie L.Chae, HeeyoungKovatcheva-Datchary, PetiaShertzer, Howard G.Chae, Soo-CheonKowalska, MalgorzataShi, QiangChan, JulieKoyama, YuShi, ZuminChan, MariaKozlowski, Michael R.Shimizu, MasahitoChan, Shun-WanKozyrskyj, Anita L.Shin, Andrew C.Chand, SourabhKrautwurst, DietmarShin, Chan YoungChang, Eugene B.Krebs, NancyShipley, PaulChang, Fang-RongKreider, RichardShirakawa, HitoshiChapwanya, AspinasKristiansen, Anne L.Shirin, ZiaeiCharumathi, SabanayagamKroeker, Karen I.Shoenfeld, YehudaChatzigeorgiou, AntoniosKrol, EwelinaShypailo, Roman J.Chaung, Hso-ChiKroll, Martin H.Sieber, FritzChen, Chun-JungKroon, PaulSiekmann, JonathanChen, Ee SinKruger, Maria J.Sievenpiper, JohnChen, Fu-AnKrul-Poel, Yvonne H.Sigounas, GeorgeChen, GuanKubena, KarenSijben, JohnChen, GuangpingKubina, RobertSilano, MarcoChen, GuoxunKubota, NaotoSilswal, NeerupmaChen, JiezhongKujawska, MałgorzataSilverberg, Donald S.Chen, XinhuaKuk, Jennifer L.Sim, Aaron Y.Chen, Yuh-FungKulozik, UlrichSim, KyraChengwen, SunKundu, Joydeb KumarŠimek, PetrCherkaoui-Malki, MustaphaKuo, Ping-ChungSimmen, FrankChey, William D.Kurdowska, Anna K.Simmer, KarenChi, YulingKurth, SalomeSimon, EdurneChiarioni, GiuseppeKuwahara, KeisukeSimsek, SuatChiba, HitoshiKwak, Chung ShilSingh, Mahendra PratapChiefari, EusebioKwiatkowska, KatarzynaSingh, NagendraChien, Yi-WenKwun, In SookSingh, Udai P.Chiesa, ScottKyrø, CecilieSingh, Ugra S.Chilibeck, PhilLa Mendola, DiegoSinghal, VibhaChiou, Wen-feiLa Vieille, SébastienSinha-Hikim, IndraniChiou, Ya-LingLac, AndrewSiniscalco, DarioChirumbolo, SalvatoreLachenmeier, DirkSint, Tin TinChiu, Wan-ChunLaferrere, BlandineSipos, FerencCho, Youn-OkLaguna, Joan-CarlesSirdah, MahmoudChoi, Myung-SookLai, Chao-QiangSiristatidis, CharalamposChoi, Wan SungLai, Jun S.Sjogren, KlaraChrismas, BrynaLaitinen, KirsiSkaaby, TeaChristian, ParulLam, Tram KimSkau, Jutta K.H.Christides, TatianaLam, Yan Y.Skeie, GuriChristofidou-Solomidou, MelpoLamacchia, CarmelaSkuladottir, Gudrun V.Chrousos, GeorgeLambert, ElisabethSluik, DiewertjeChu, DachenLambert, Joshua D.Sluimer, JudithChu, ShidongLand, Mary-AnneSmart, NeilChudek, JerzyLandberg, RikardSmedslund, GeirChun, Ock K.Landete, José MariaSmid, Scott D.Chung, Louisa M.Y.Landi, FrancescoSmith, ClaireChyou, Wei WeiLane, DariusSmith, GordonCibulskis, CatherineLang, Undine E.Smith, JohnEric W.Ciccone, Marco MatteoLanou, Amy JoySmith, Kylie J.Cicerale, SaraLarqué, ElviraSmith, MoiraCicero, ArrigoLarrosa, MarSmoliga, JamesCiclitera, PaulLarsen, BriannaSmyth, PeterCione, ErikaLasekan, John B.Snih, Soham A.l.Clarke, KieranLass, AchimSoare, AndreeaClaudia, VetraniLassek, WilliamSoares, MarioClavel, ThomasLatruffe, NorbertSobczyńska-Malefora, AgataCleary, Margot P.Lavoie, Jean-claudeSoedamah-Muthu, Sabita S.Clegg, Miriam E.Layman, DonSoenen, StijnClemens, RogerLazzeri, GiacomoSolah, Vicky A.Clere, NicolasLean, Michael E.J.Soliman, Karam F.A.Clifton, PeterLederer, Eleanor D.Song, Won O.Clugston, RobinLee, ElaineSong, YoonjuCoan, Philip M.Lee, Sun Y.Sop, Marie M.K.Codling, KarenLee, Sun-OkSouidi, MaamarCoe, Christopher L.Lee, Tzong-shyuanSoulage, ChristopheCoëffier, MoïseLee., Warren T.K.Sourij, HaCohen, GeraldLeech, Rebecca M.Souyri, MicheleCohen, Jacqueline M.Leffler, Daniel A.Spaeth, AndreaCohen, RonaldLehtisalo, JenniSpano, GiuseppeColeman, HelenLeiherer, AndreasSparrow, JanetColitti, MonicaLemay, Danielle G.Sparvoli, FrancescaColleran, Heather L.Lenaerts, KaatjeSpégel, PeterColson, NatalieLentjes, MarleenSpence, J. DavidColumbus, Daniel A.Leombruni, PaoloSpiegler, JulianeColussi, GianLucaLeone, SerenaSprague, MatthewCombet, EmilieLesgards, Jean-FrançoisSpriet, LawrenceComelli, Elena M.Lesmes, UriSpuch, CarlosComerford, Kevin B.Letizia, ClaudioSreelatha, S.Comino, IsabelLeung, CindySrigley, Cynthia T.Conceição, EvaLeung, Ricky Yuet-KinStadlbauer, VanessaCondo, DominiqueLeung, Yuk-manStadler, Diane D.Conklin, AnnalijnLever, MichaelStaege, Martin S.Conlon, CathrynLevy, LouisStagg, AndrewConrad, Zach S.Levy, YishaiStagkos, DimitriConroy, DeirdreLewinska, AnnaStandl, MarieConterno, LorenzaLi Volti, GiovanniStangl, Gabriele I.Conti, PioLi, DuoStanley, RogerCooke, MattLi, JiaStanstrup, JanCooper, Jamie A.Li, MilingStaples, CharlesCooperstone, Jessica L.Li, MingStark, KenCoppedè, FabioLiaset, BjørnStarling, Anne P.Coppola, GianlucaLiberato, Selma C.Steegers - Theunissen, RegineCordero, PaulLibro, RosalianaStefanidou, Maria E.Cordle, Chris T.Lichtenstein, AliceStehle, PeterCorella, DoloresLieberman, Harris R.Steinbrenner, HolgerCorish, ClareLiem, GieSteiner, Jennifer L.Corniola, RikkiLillehoj, Hyun S.Steinhoff, UlrichCornish, StephenLillioja, StephenStengel, AndreasCorrea, Marcos BrittoLim, SooStephanou, AnastasisCorsaro, CarmeloLin, EugeneStewart, MariaCorvaglia, LuigiLin, Pao-hwaStewart, Michael W.Costa, Pablo B.Lin, Ping-TingStilli, DonatellaCostagliola, CiroLin, Shyh-HsiangStockmann, ChristianCostelli, PaolaLin, Yu-HsinStokes, Caroline S.Costello, RebeccaLindeberg, StaffanStoner, Gary D.Cravero, Maria CarlaLindsay, AngusSt-Onge, Marie-PierreCrenn, PascalLingwood, BarbaraStookey, JodiCrichton, GeorginaLiou, Ying-mingStote, KimCristina, Gastalver-MartínLister, NatalieStough, ConCroft, KevinLitchford, Mary D.Stout, JeffCrosbie-Watson, RachelleLitonjua, AugustoStrandvik, BirgittaCrowe, SheilaLitonjua, Augusto A.Strasser, BarbaraCrume, TessaLittle, Jonathan P.Stratos, IoannisCrystal, LimLittle, Tanya J.Strazzullo, PasqualeCui, XiaoyingLiu, AipingStreisand, RandiCulig, ZoranLiu, JianStudzinski, George P.Culot, MaximeLiu, Pei-YangSu, Chun-LiCumming, Kristoffer T.Liu, Tie FuSu, JosephCuppari, LilianLiu, ZhenhuaSuarez-Varela, Maria M.Cureton, Pamela A.Llaneza-Suarez, DavidSubar, Amy F.Currell, KevinLo Monte, Attilio I.Suckow, Arthur T.Cutuli, DeboraLo Muzio, LorenzoSui, ZhixianCzaja-Bulsa, GrażynaLo, Hui-ChenSukkar, SamirD’Archivio, MassimoLo, Wai-KeiSuliburska, JoannaD’El-Rei, JeniferLockshin, LarrySullivan, Sheila CoxDacks, Penny A.Lof, MarieSun, GuangDahech, ImenLoffing, JohannesSun, Hui SunDahl, CecilieLoffredo, LorenzoSun, MingkuanDaiello, Lori A.Lombó, FelipeSung, Mi-KyungDailey, Megan J.London, EdraSurai, PeterDal Monte, MassimoLongardt, Ann C.Suzuki, TakuyaDalamaga, MariaLópez, Inés VelascoSuzuki, Yuichiro J.D'Alessio, Patrizia A.Lopez, VictorSuzutani, TatsuoDamsgaard, Camilla T.Lopez-Garcia, EstherSvendsen, Ida S.D'Angelo, StefaniaLophatananon, ArtitayaSverker, AnnetteDanielson, Kirstie K.Lorenz, LailaSweeney, Mary RoseD'Argenio, GiuseppeLouie, JimmySzekeres, ThomasDarling, Andrea L.Louis, PetraSzopa, AgnieszkaDarnton-Hill, IanLövestam, ElinTaal, Maarten W.Das, Undurti N.Lowe, NicolaTajmir-Riahi, Heidar A.Dasarathy, SrinivasanLu, Chi-HuaTakachi, RibekaDashti, HassanLu, Kuo-ChengTakahisa, KanekiyoDavidson, MayerLu, WenhuaTakaya, JunjiDavison, Karen M.Luévano-Contreras, ClaudiaTakechi, RyusukeDawodu, AdekunleLukaski, HenryTakeda, EijiDawson, John A.Lukaszuk, JudithTakimoto, HidemiDe Cássia Aquino, RitaLundberg, JonTakkunen, MarkusDe Castro Barbosa, ThaisLundberg, TommyTako, EladDe Gaetano, GiovanniLundin, KnutTalsma, Elise F.De Klerk, Nicholas H.Lundström, PatrikTan, DunxianDe Kreutzenberg, SaulaLutz, JensTanaka, MasakiDe La Barca, Ana M.C.Lyon, GrahamTaneja, VeenaDe La Monte, SuzanneLyons, BeverlyTangney, Christy C.De La Parra, ColumbaLyons, Paul E.Tappia, Paramjit S.De Lourdes Pereira, MariaMa, YiyiTappy, LucDe Lourdes Samaniego-Vaesken, MaríaMa, YongjieTarantino, GiovanniDe Martino, LauraMa, YunshengTaranu, IoneliaDe Medina, F. SánchezMa, Zheng FeeiTarapore, PheruzaDe Paepe, BoelMacDonald-Wicks, LesleyTashiro, HirotakaDe Pascual-Teresa, SoniaMacedo, RodrigoTauler, PedroDe Pascual-Teresa, SoniaMackerras, Dorothy E.M.Taylor, CarolineDe Santo, CarmelaMacklon, Nick StephenTaylor, SteveDe Souza, RussellMacpherson, HelenTchernof, AndréDe Steur, HansMadar, Ahmed A.Te Morenga, LisaDe Vries, JeanneMadhukar, BurraTeas, JaneDe Wild, Victoire W.T.Madimenos, FeliciaTeasdale, ScottDeGraffenried, Linda A.Maffeis, ClaudioTemme, Elisabeth H.M.Dekker, MarloesMafra, DeniseTemple, Jennifer L.Del Rio, DanieleMagee, Pamela J.Temple, Norman J.Delarue, JacquesMaglio, MariantoniaTeodoro, Anderson J.Deldicque, LouiseMagnussen, Costan G.Teske, Jennifer A.Delisle, HélèneMaher, PamelaTestai, LaraDella Torre, SaraMahmud, FaridTester, EmmaDellavalle, Diane M.Mahoney, Sara E.Thakur, Raghu RajDenney, LiyaMai, VolkerThalacker-Mercer, AnnaDepoortere, IngeMaillard, VirginieThaler, JoshuaDesbrow, BenMakela, JohannaTheodora, PsaltopoulouDetzel, PatrickMaki, KevinThèvenod, FrankDeutz, Nicolaas E.Malavolta, MarcoThielecke, FrankDevaraj, SrideviMalisova, OlgaThiem, UrsulaDevlin, AngelaMalkova, DaliaThomas, DavidDey, MoulMallard, Simonette R.Thomas, DianaDezaki, KatsuyaMallidou, AnastasiaThomas, Lynn K.Dhar, AnimeshManary, MarkThomas, TravisDharmasena, SenarathManca, Maria LetiziaThompson, Campbell H.Di Daniele, NicolaManco, MelaniaThompson, Frances E.Di Felice, GabriellaMandalari, GiuseppinaThompson, HenryDi Iorio, BiagioMann, Michelle CatherineThomsen, HaukeDi Lorenzo, CherubinoManni, AndreaThomsen, Jesper S.Diaz, FranciscoMantell, Lin L.Threapleton, DEDickerson, RolandManti, SaraThunnissen, ErikDickinson, JaredMantovani, GiovanniThurnham, DavidDiel, PatrickMarangoni, FrancaTimmerman, Kyle L.Diep Yeung, Cassandra S.Marcus, Carole L.Tinker, Sarah C.Dierkes, JuttaMargolis, LeeTitgemeyer, EvanDietrich-Muszalska, AnnaMaria José, Rodriguez LagunasTitorenko, Vladimir I.Dillon, E. LicharMarin, Daniela E.Tiwari, AmitDimas, KonstantinosMarini, ElisabettaTokuyama, KumpeiDiNicolantonio, James J.Marini, Juan C.Tomaro-Duchesneau, C.Dipnall, Joanna F.Marino Donangelo, CarmenTomasello, GiovanniDo Rosário Bronze, MariaMarion-Letellier, RachelTondo, LeonardoDoh, Kyung-OhMarlow, GarethTong, IrisDoig, GordonMarra, MaurizioTonstad, SerenaDolinsky, Vernon W.Marriott, Bernadette P.Tornberg, EvaDomenicotti, CinziaMartin, CamiliaTorres, Márcia R.S.T.Donini, Lorenza MariaMartin-Calvo, NereaTorres, SusanDost, TurhanMartínez, HomeroTovoli, FrancescoDou, Q. PingMartinez, JessicaToxqui, LauraDoulias, Paschalis-ThomasMartinez, KristinaTraber, MaretDraijer, RichardMartinez, VicenteTraina, GiovannaDreher, MarkMartínez-Augustin, OlgaTran, ChristelDrevon, Christian A.Martinez-Gonzalez, Miguel A.Treesukosol, YadaDrougard, AnneMarventano, StefanoTrehan, IndiDu, MinMarverti, GaetanoTremblay, AngeloDuarte Almeida, Joaquim M.Marzocco, StefaniaTresguerres, JesúsDube, JohnMarzullo, PaoloTriunfo, StefaniaDuckworth, Lauren C.Mascherini, GabrieleTroen, AronDueñas, MontserratMasilamani, MadhanTroesch, BarbaraDuez, PierreMaslova, EkaterinaTrovato, Francesca M.Dugo, GiacomoMassot, MalenTrovato, GuglielmoDullaart, Robin P.F.Mastrocola, RaffaellaTrovato, Guglielmo M.Dunford, ElizabethMasuo, KazukoTsai, YingChiehDuntas, LeonidasMaszczyk, AdamTsao, FrancisDupont, ChristopheMateu-de Antonio, JavierTsiani, EvangeliaDupont, JoëlleMathus-Vliegen, Elisabeth M.H.Tsin, AndrewDurante, WilliamMatias, CatarinaTsopmo, ApollinaireDwyer, KarenMatsumoto, HiroyukiTsuji, PetraEastman, CreswellMatsuzaki, KeiichiTsuruya, KazuhikoEconomopoulou, PanagiotaMaukonen, MirkkaTsutsumi, RieEdwards, Kate M.Mauriege, PascaleTufarelli, VincenzoEfremov, Sergey M.Mcauliffe, FionnualaTulipano, GiovanniEhret, Georg B.McBean, GethinTung, Tao-HsinEichler, KlausMcCall, Charles E.Tur, Josep AntoniEilertsen, Karl-ErikMcClung, James P.Turner, DanEkmekcioglu, CemMcCrickerd, KeriTurner, MarkEkuni, DaisukeMcCrory, Megan A.Turner, R. ScottEldridge, Alison L.McDaniel, JohnTurrini, EleonoraEleftheriadis, TheodorosMcElroy, Steven J.Tuuri, GeorgiannaElgueta, RaulMcFarlin, Brian K.Twigg, StephenElinder, Carl GustafMcGlory, ChrisTyagi, Amit K.Elisabet, ForsumMcGowan, Ciara A.Tylavsky, Frances A.Elli, LucaMcJarrow, PaulTyler, ChurchwardElliott, Michael B.McKenna, Malachi J.Tziomalos, KonstantinosEmbleton, NicholasMcKenzie, MikeUberti, FrancescaEmmert, SteffenMcKinley, Michael J.Uehara, MarikoEmwas, AbdelhamidMcLean, RachaelUemura, HirojiEngelen, Mariëlle P.K.J.Mcmahon, EmmaUjcic-Voortman, JoanneEngle-Stone, ReinaMcMurray, FionaUllrich, ReinerErikson, KeithMcNaughton, Lars R.Umegaki, HiroyukiErkkilä, Arja T.McNulty, HeleneUnick, Jessica L.Escobar, Matthew A.McPherson, Nicole O.Urashima, TadasuEspinosa-Martos, IreneMcTernan, PhilipUrrestarazu, ElenaEstruch, RamonMears, Stephen A.Vafeiadi, MarinaEvans, GethinMedina, F. XavierVago, RiccardoEyles, Darryl W.Medler, Kathryn F.Valdés-Ramos, RoxanaFabroni, SimonaMeier, PaulaValentinuzzi, FabioFacal, DavidMeli, RosariaValenzuela, RodrigoFadda, RobertaMeliker, Jaymie R.Van Baak, Marleen A.Fairchild, TimothyMellberg, CarolineVan Ballegooijen, HanneFairweather-Tait, SusanMendez, RosaVan Bodegraven, Ad A.Fallows, StephenMerino, GraciaVan Den Ende, WimFan, XiuchengMerino, RamónVan Der Kamp, Jan WillemFang, TechaoMerle, Bénédicte M.J.Van Der Voet, GijsbertFanos, VassillosMero, AnttiVan Dijk, Suzanne C.Fanti, PaoloMerrill, Rebecca D.Van Goudoever, Johannes B.Farhat, GraceMessner, BarbaraVan Hoorenbeeck, KimFaria, AnaMetzinger, LaurentVan Loon, LucFatouros, Ioannis G.Meyer, Katie A.Van Loon, Luc JcFaust, Andrew C.Meyerholz, DavidVan Raaij, JoopFayet-Moore, FlaviaMeyre, DavidVan Schothorst, Evert M.Fedele, FrancescoMian, CaterinaVanamala, Jairam K.P.Feinman, Richard D.Miccadei, StefaniaVance, David E.Fenko, AnnaMichael, ConlonVaquero, PilarFenton, TanisMichels, NathalieVarela-Moreiras, GregorioFernandes, Alexandra R.Mielgo-Ayuso, JuanVashist, Yogesh K.Fernandes, Maria HelenaMiettinen, MaijaVasiljevic, TodorFernández, IgnacioMiles-Chan, Jennifer L.Vassalle, CristinaFernandez, JoseMiller, Carla K.Vaughan, Roger A.Fernandez, Maria LuzMiller, Gary D.Vecchia, CarloFerrante, AntonioMiller, Leland V.Vecchione, CarmineFerreira, IsabelMilliron, Brandy-JoeVega, Gloria LenaFerreira, Tracie L.Millward, JoeVenn, BernardField, CatherineMineo, ChiekoVerbalis, Joseph G.Fielding, BarbaraMinihane, Anne MarieVerbeke, KristinFields, DavidMiralles, BeatrizVerd, SergioFine, James BurkeMisciagna, GiovanniVerdijk, LexFineschi, VittorioMistretta, AntonioVerduci, ElviraFinkel, Alan G.Mistry, Hiten D.Vergara, DanieleFisher, JeffreyMitchell, CameronVerheij, MarcelFiszman, SusanaMitchell, DianeVetvicka, VaclavFleenor, Bradley S.Miyata, JunVeugelers, Paul J.Flueck, Joelle L.Miyata, YasuyoshiVicente-Salar, N.Fond, GuillaumeMizuno, KatsumiVictor, Ronald G.Fong, Bertram Y.Moldzio, RudolfVidal, Cecilio J.Ford, AndrewMoliner-Urdiales, DiegoVieira-Potter, VictoriaFord, StephenMøller, U. KristineVignini, AriannaForjuoh, Samuel N.Monaci, LindaVilaplana, Amadeo G.Fort, PatriceMonjardino, TeresaVinceti, MarcoFoster, EmmaMonk, JenniferVioque, JesúsFoufelle, FabienneMonro, JohnVisioli, FrancescoFrame-Peterson, Leigh A.Montalcini, TizianaVollmer-Conna, UteFrancesco, MasoeroMonti, Daria MariaVon Hurst, Pamela R.Francino, PilarMontoya, Carlos A.Von Lintig, JohannesFrancis, HeatherMoore, DanielVon Lintig, JohannesFranco, OctavioMoore, Latetia V.Von Schacky, ClemensFrank, SašaMorabito, AntoninoVon Wright, AtteFrankenfeld, Cara L.Moran, MichaelVonk, RoelFreedman, MarjorieMoranta, DavidVoortman, TrudyFreije, José M.P.Moreno, Juan J.Voznesenskaya, AnnaFreitas, Ana C.Moreno-Indias, IsabelVucenik, IvanaFresco, PaulaMoretti, DiegoWada, JunFrestedt, Joy L.Morgante, GiuseppeWagner, Bridget K.Friel, JamesMorioka, TomoakiWagner, CarolFriel, James K.Morita, ManabuWalker, Jacqueline L.Frongillo, Edward A.Morris, AndrewWalker, Mary K.Fu, QiangMorris, Martha C.Walker, ValenciaFu, Tzu-FunMorrison, DouglasWall, BenFuchs, DietmarMoser, OthmarWallace, DavidFuge, RonMounier, CatherineWallace, TaylorFujimori, KoMousa, Shaker A.Walton, JanetteFukui, MichiakiMuggeridge, DavidWang, Chi ChiuFukumoto, YoshihiroMukhopadhyay, ParthaWang, DavidFullston, TodMukhtar, HasanWang, Feng-ShengFunato, HiromasaMuldoon, Matthew F.Wang, HongFung, Ellen B.Mulhern, Maria S.Wang, Hsueh-FangFuster-Parra, PilarMüller, Manfred JamesWang, JiaweiGahche, JaimeMulligan, Angela A.Wang, JingGallant, Kathleen M.H.Murata, MarikoWang, JunGalleano, MonicaMurphy, CaoileannWang, Ling ZhiGambon, Dien L.Murphy, Michelle M.Wang, Ming-DongGammon, MarilieMurray, JosephWang, Peizhong PeterGandini, SaraMurthi, PadmaWang, TingGandy, JoanMusch, MarkWard, LeighGangula, PanduMutoh, MichihiroWard, MaryGanini, DouglasMutti, ElenaWardenaar, Floris C.Ganio, Matt S.Myneni, AjayWarner, MarcellaGarden, FrancesNa, MuziWarren, FredGardner, DavidNader, Nader D.Watanabe, KenichiGarinis, GeorgeNagai, YoshinoriWatkins, Adam J.Garnett, SarahNagatoishi, SatoruWatkins, Colleen MarieGassler, NikolausNair-Shalliker, VisaliniWatson, AndrewGazouli, MariaNaito, YoshiroWatt, PeterGeisler, CorinnaNaito, YujiWax, BenjaminGellerman, WernerNakajima, YoshihiroWaxman, JoshuaGentili, SheridanNakano, YuyaWeaver, Connie M.Georgiev, VasilNamork, EllenWebb, Andrew J.Georgoulis, MihalisNansel, TonjaWebster, JacquiGerber, Leonard E.Napoli, JosephWegehaupt, FlorianGevers, Dorus W.M.Nardo, BrunoWeghuber, DanielGhalib, Raza MuradNascimento, Gustavo G.Weickert, Martin O.Gharaibeh, BurhanNathan, NicoleWeijs, Peter J.M.Ghirri, PaoloNavarrete-Muñoz, Eva M.Weir, TiffanyGiaginis, CostantinosNavarro, Carlos J.G.Weiss, ScottGiakoustidis, Dimitrios E.Navarro-Alarcon, MiguelWelbourne, RichardGiannì, Maria LorellaNavas-Carretero, SantiagoWelch, AilsaGianotti, LucaNebot Valenzuela, ElenaWelch, Robert W.Giapros, VasileiosNegro, MassimoWeldon, Douglas A.Gibbs, JennaNeilson, AndrewWelsh, Jean A.Gibson, Alice A.Nestel, PaulWelsh, MichaelGibson, Peter R.Newby, RuthWen, LiGibson, SigridNewnham, EvanWeng, Ching-FengGidley, MikeNicastro, Holly L.Wenhold, Friedeburg A.M.Giera, MartinNichols, Buford L.wesson, DonaldGil-Campos, MercedesNichols, Erin K.West, KeithGiles, Grace E.Nichols, Nancy N.Westmark, Cara J.Gill, ChrisNicklas, TheresaWeyermann, MariaGill, Simone V.Nielsen, ForrestWhisner, CorrieGiltay, ErikNieman, DavidWhisner, Corrie M.Giordano, MauroNiewold, TheoWhitaker, Hayley C.Giuberti, GianlucaNikolaidis, Michalis G.White, JohnGłąbska, DominikaNiles, Richard M.Whiting, Susan J.Glade, Michael J.Nishikawa, YoshikazuWie, Myung-BokGlahn, RayNobile, VincenzoWieser, SimonGletsu-Miller, NanaNogueira, Rossana C.Wightman, Emma L.Godek, Sandra FowkesNolan, JohnWillems, MarkGoel, NamniNomura, YoshihiroWilliams, BarbaraGoer, MaureenNørskov, Natalja P.Williams, ElizabethGoh Boon Bee, GeorgeNorthstone, KateWilliamson, DavidGold, Paul E.Norval, MaryWilliamson, KevinGoletzke, JaninaNybacka, SannaWilling, BenjaminGollapudi, SastryO’Grady, Thomas J.Willoughby, DarrynGombart, AdrianO’Hagan, CiaraWilson, TedGómez-Ambrosi, JavierObana, AkiraWinham, Donna M.Gómez-Cabello, AlbaObeid, RimaWinichagoon, PattaneeGoni, FelixOberhelman, SaraWirth, StefanGonzalez, MichaelObrador, ElenaWise, Paul M.González, SoniaO'Brien, Sarah H.Witte, VeronicaGonzález-Correa, SoniaO'Connell, ThomasWitt-Enderby, PaulaGoodall, StuartO'Connor, LauraWłodarek, DariuszGoossens, Gijs H.Ogan, DanaWolfe, Robert R.Gosby, Alison K.Ogino, ShujiWong, Grace Lai–HungGosetti, FabioOhfuji, SatokoWong, Jyh EiinGostner, Johanna M.Ohta, YoshijiWoo, Jessica GrausGouveia, Élvio R.Okada, FutoshiWoods, Alisa G.Gow, Megan L.O'kane, GabrielleWoolf, KathleenGoya, LuisOkazaki, RyoWu, Chun-ChiGracia, IgnacioOliveira, Paula A.Wu, Ting-FengGraham, Dan J.Oliveras-López, María-JesúsWung, Being-SunGranfors, MichaelaOlmedilla-Alonso, BegoñaXia, TingtingGrange, Robert W.Olsen, NannaXie, ChenGrant, WiliamOlza, JosuneYabe, DaisukeGrasser, Erik KonradOmoruyi, FelixYadav, HariomGreen, Benjamin PaulOms-Oliu, GemmaYamamoto, NorioGreen, CharlotteOnufrak, StephenYamasaki, KeishiGreen, TimOrfila, CarolineYan, LinGreer, Frank R.Ormsbee, MichaelYang, FajunGregersen, SørenO'Rorke, MichaelYang, Hsin-YiGresham, EllieOrtiz, AlbertoYang, Kuender D.Grider, ArthurOsborne, BrennaYang, WoongMoGrieger, Jessica A.Oshima, TadayukiYang, YeGriffin, IanOster, HenrikYao, SongGriffiths, Helen R.Ota, TsuguhitoYde, Christian ClementGrimm, Marcus O.W.Otsuka, YuzuruYedery, Roshan D.Grootaert, CharlotteOude Griep, LindaYeh, Kuo-WeiGrosso, GiuseppeOuziel, RomyYiannakopoulou, EugeniaGu, HarvestØverby, Nina C.Yin, Mei-chinGu, YianOwen, JonathanYokoi, KatsuhikoGuandalini, StefanoOz, Helieh S.Yoo, Hwa-SeungGuéant, Jean-LouisOzaki, C. KeithYoshida, HiroshiGuenther, Patricia M.Ozen, MustafaYoung, Jette F.Gugliucci, AlejandroPabalan, Noel A.Young, WayneGuimón, JoséPaddon-Jones, DouglasYu, SolomonGulati, VandanaPaech, GemmaYu, Yan PingGunaratna, Nilupa S.Pagano, Mario AngeloYui, KunioGunnarsdottir, IngibjörgPage, Kathleen A.Yung, RaymondGuo, FenghaiPagonopoulou, OlgaZajac-Kaye, MariaGupta, AnshuPalacios, CristinaZang, LiqingGupta, SubashPallauf, KathrinZanini, BarbaraGustafsson, Jan-ÅkePalmer, Amanda C.Zanke, BrentGutiérrez-Juárez, RogerPalmer, MichelleZara, VincenzoHa, Ki-TaePalumbo, CarlaZavattari, PatriziaHaase, HajoPanahi, ShirinZemel, MichaelHaboubi, NadimPanaro, Maria AntoniettaZeng, HuaweiHafekost, KatherinePantano, Kathleen JoanZhang, DeqiangHajduch, EricPaoli, AntonioZhang, GeHalade, GaneshPaolocci, NazarenoZhao, LeiHaldar, SumantoPapadopoulos, Nikolaos G.Zhao, YunHalldorsson, Thorhallur IngiPark, Gyeong-HunZheng, MiaobingHalmos, EmmaPark, SohyunZhu, MeijunHambidge, MichaelPark, WansuZiegler, EkhardHammerling, UlrichPark, YongsoonZilli, GislaineHamner, HeatherParkar, Shanthi G.Zoetendal, ErwinHampel, DanielaParker, HelenZortea, KarineHancock, Chad R.Parker, Leslie A.Zosky, Graeme R.Handel, Mina N.Parlesak, AlexandrZuccotti, Gian V.Hannibal, LucianaParletta, NatalieZughaier, Susu M.Hansen, MetteParnell, Jill A.Zuhl, MicahHanson, CorrineParr, EvelynZuo, LiHao, LeiParrettini, SaraZuo, YuegangHardiman, GaryPartearroyo, TeresaZwart, SaraHardman, W. ElainePase, Matthew P.Zwetsloot, KevinHaring, BernhardPastor Vicedo, Juan Carlos


